# A Rare Case of Congenital Simple Cystic Ranula in a Neonate

**DOI:** 10.1155/2013/841930

**Published:** 2013-09-04

**Authors:** Gautam Bir Singh, Anil K. Rai, Rubeena Arora, Sunil Garg, Pooja Abbey, Shailaja Shukla

**Affiliations:** ^1^Department of Otorhinolaryngology, Lady Hardinge Medical College & Associated Hospitals, Shaheed Bhagat Singh Marg, New Delhi 110001, India; ^2^Department of Radiology, Lady Hardinge Medical College & Associated Hospitals, Shaheed Bhagat Singh Marg, New Delhi 110001, India; ^3^Department of Pathology, Lady Hardinge Medical College & Associated Hospitals, Shaheed Bhagat Singh Marg, New Delhi 110001, India

## Abstract

Congenital ranula in a neonate is an uncommon occurrence. We present one such case of the said lesion where the clinical presentation and management were found to be interesting, hitherto unreported in the medical literature. This clinical record also reviews the scant medical literature on congenital ranula in neonates.

## 1. Introduction

Ranulas are cystic dilatations in the floor of the mouth and a result of obstruction of one of the sublingual salivary glands. It is basically a retention cyst. Ranula may be congenital or acquired. Congenital ranula in newborn infants is a rarity [1–3] and thus there is a marked paucity of the literature on the cited subject. With this background we present one such case of congenital ranula in a neonate which was symptomatic and required surgical management. To the best of our knowledge, such a case has not been previously reported in the medical literature. With this case, we illustrate a rare entity that is present in an extremely rare manner.

## 2. Case Record

**Figure 1:**
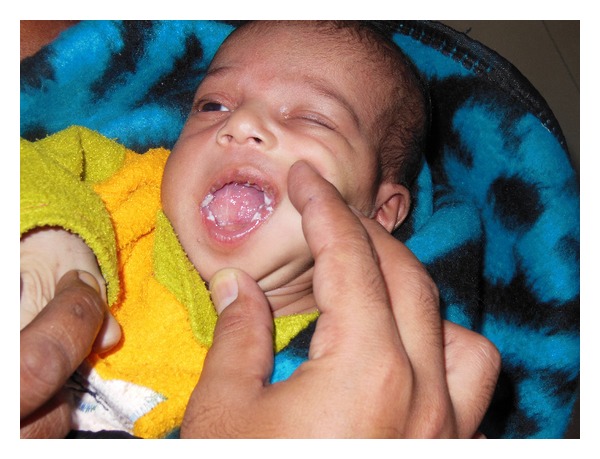
Clinical photograph of the patient with congenital ranula.

**Figure 2:**
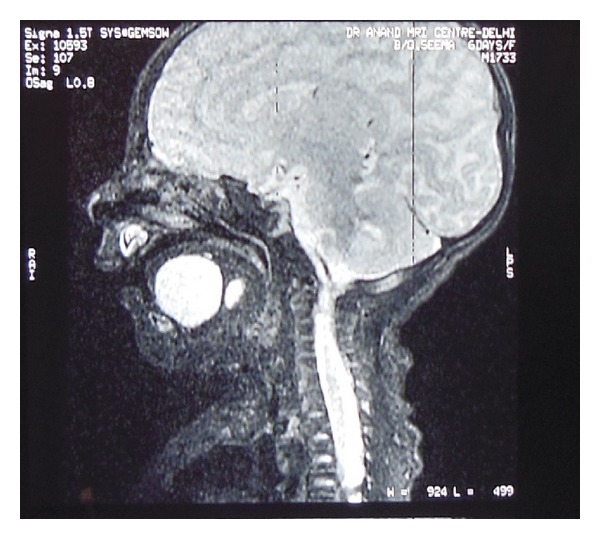
MRI scan: midline sagittal fat suppressed T2 weighted image showing the cystic lesion above the mylohyoid with no extension.

**Figure 3:**
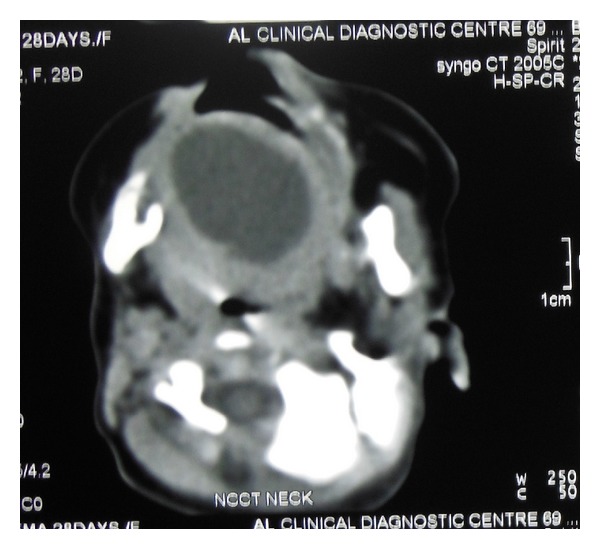
CT scan showing the cystic lesion in the axial cut.

**Figure 4:**
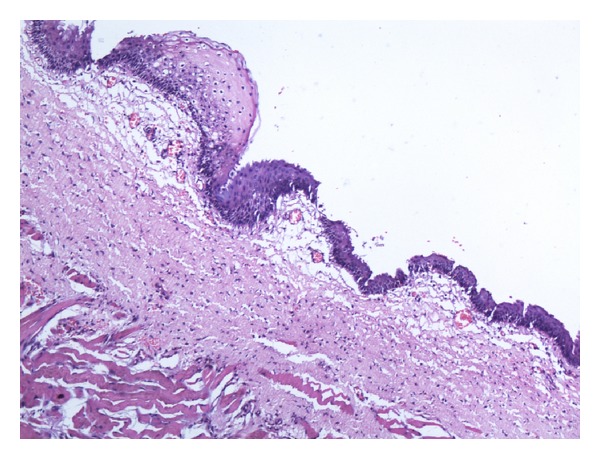
Cyst wall lined by stratified squamous epithelium with presence of mucus glands within the wall.

A full-term male neonate was referred to our teaching hospital 20 days after birth with a swelling in the sublingual region since birth (Figure 1). The swelling had increased over a period of time causing difficulty in feeding and noisy breathing leading to failure to thrive. The differential diagnosis of lymphatic malformations, teratoma, dermoid cyst, and thyroglossal duct cysts was considered. But finally a diagnosis of “congenital ranula” was concluded upon clinical examination and radiological investigations [2]. The CT scan and MRI scan delineated a well-defined cystic lesion (2.65 × 1.7 × 2.21 cm) in the midline of the floor of mouth above the level of mylohyoid (Figures 2 and 3). As the ranula was symptomatic and more than 1 cm in diameter, a complete excision of the ranula was done [1, 4]. Post-op period was uneventful, with no recurrence for the last 9 months. The histopathology of the excised lesion revealed a simple cyst lined by stratified squamous epithelium with the presence of mucus glands within the wall consistent with simple cystic ranula (Figure 4).

## 3. Discussion

It would be prudent to note that ranulas are of two types [5]: the uncommon simple cystic ranula which represents partial obstruction of the distal end of sublingual gland duct or perhaps other minor salivary gland tissue in the floor of mouth, and it is usually less than 1 cm in diameter. And the other type is the common mucus extravasation pseudocyst as a result of the escape of mucus through a ruptured sublingual duct into the adjacent connective tissue. They are not true cysts and are lined by granulation tissue. However we recorded a large simple cystic ranula of more than 2 cm in diameter in this neonate. In addition, congenital ranulas are usually asymptomatic and are resolved with time. Most likely the explanation for resolution would be a rupture as a result of feeding [3]. On the contrary, we had a symptomatic congenital ranula in a neonate.

Most of these cases are diagnosed clinically at the time of birth. CT scan and MRI scan are useful investigations which help to delineate the lesion and formulate a definitive diagnosis as clearly evident from this case report as well [2]. An MRI scan may be regarded as a gold standard as it not only gives high resolution images, determines precise location and content of the lesion but also enhances the differentiation of ductal atresia from duplication anomalies of ductal system [6]. Interestingly, the review of the recent literature on the cited subject reveals that these congenital lesions can now be diagnosed prenatally by ultrasonography and an EXIT (ex-utero intrapartum treatment) regime may be followed for treatment in such cases [7, 8].

The treatment protocol for paediatric ranula is still controversial. The medical literature recommends observation for asymptomatic lesions as spontaneous resolution does occur in some cases. Several methods for treatment have been reported [2, 4, 5]: aspiration, cryosurgery, marsupialization, placement of silk suture in the dome of ranula, and excision of the cyst with or without sublingual gland excision. The case in focus makes a strong point for surgical excision of symptomatic congenital ranula to prevent its recurrence. Even if later on these excised lesions are pathologically diagnosed as extravasation pseudocyst the chances are that the offending mucous gland has been removed. Further, it would also be imperative to note that psuedocysts have no epithelial lining; hence, marsupialization would inevitably lead to recurrence. Finally, the authors would like to emphasise that in any lesion more than 1 cm in diameter, plunging ranula or cervical ranula, origin from sublingual gland is to be presumed and, therefore, surgical excision with the sublingual gland is the treatment of choice [5].

In a massive internet search using PubMed/MEDLINE services, authors could not find any case of symptomatic congenital simple cystic ranula in a neonate as reported herein. The previous three cases reported in neonates were asymptomatic, and in one case needle aspiration was done, while the other two cases have resolved spontaneously over a period of time [9, 10]. Thus, from the aforesaid discussion we conclude that (i) congenital ranula can be symptomatic in a neonate, (ii) an MRI scan helps to clinch the diagnosis, (iii) such lesions should be treated surgically to prevent recurrence.

In summary, the rarity of this lesion in neonates and its atypical clinical presentation and management make this case report unique and thus prompted us to share our clinical experience with the medical fraternity.
